# Definition of common carotid wall thickness affects risk classification in relation to degree of internal carotid artery stenosis: the Plaque At RISK (PARISK) study

**DOI:** 10.1186/s12947-017-0097-4

**Published:** 2017-04-04

**Authors:** J Steinbuch, AC van Dijk, FHBM Schreuder, MTB Truijman, J Hendrikse, PJ Nederkoorn, A van der Lugt, E Hermeling, APG Hoeks, WH Mess

**Affiliations:** 1 Biomedical Engineering, Cardiovascular Research Institute Maastricht, Maastricht University, Maastricht, The Netherlands; 2 Radiology, Erasmus Medical Center, Rotterdam, The Netherlands; 3 Neurology, Erasmus Medical Center, Rotterdam, The Netherlands; 4 Radiology, Maastricht University Medical Center, Maastricht, The Netherlands; 5 Clinical Neurophysiology, Maastricht University Medical Center, PO Box 5800, 6202 Maastricht, AZ The Netherlands; 6 Neurology, Maastricht University Medical Center, Maastricht, The Netherlands; 7 Radiology, University Medical Center Utrecht, Utrecht, The Netherlands; 8 Neurology, Academic Medical Center, Amsterdam, The Netherlands

**Keywords:** Atherosclerosis, Stenosis, Carotid IMT, Ultrasound, Carotid artery imaging

## Abstract

**Background:**

Mean or maximal intima-media thickness (IMT) is commonly used as surrogate endpoint in intervention studies. However, the effect of normalization by surrounding or median IMT or by diameter is unknown. In addition, it is unclear whether IMT inhomogeneity is a useful predictor beyond common wall parameters like maximal wall thickness, either absolute or normalized to IMT or lumen size. We investigated the interrelationship of common carotid artery (CCA) thickness parameters and their association with the ipsilateral internal carotid artery (ICA) stenosis degree.

**Methods:**

CCA thickness parameters were extracted by edge detection applied to ultrasound B-mode recordings of 240 patients. Degree of ICA stenosis was determined from CT angiography.

**Results:**

Normalization of maximal CCA wall thickness to median IMT leads to large variations. Higher CCA thickness parameter values are associated with a higher degree of ipsilateral ICA stenosis (*p* < 0.001), though IMT inhomogeneity does not provide extra information. When the ratio of wall thickness and diameter instead of absolute maximal wall thickness is used as risk marker for having moderate ipsilateral ICA stenosis (>50%), 55 arteries (15%) are reclassified to another risk category.

**Conclusions:**

It is more reasonable to normalize maximal wall thickness to end-diastolic diameter rather than to IMT, affecting risk classification and suggesting modification of the Mannheim criteria.

**Trial registration:**

Clinical trials.gov NCT01208025.

## Background

An irregular intima-media thickness (IMT) of the common carotid artery (CCA) is indicative for atherosclerotic burden [1, 2] and hence, might be a useful predictor in risk assessment. In a vascular diseased patient population CCA-IMT irregularity is associated with nearby atherosclerosis [2]. Furthermore, in symptomatic patients high CCA-IMT irregularity is associated with a higher degree of stenosis of distal plaques [1] and is more prominent in symptomatic than in asymptomatic subjects [3].

However, as previously discussed by Bots et al. [4], it remains unclear whether IMT irregularity itself is a useful predictor in addition to maximal IMT. It has been shown that after adjustment for coronary risk factors the combined IMT irregularity of CCA, bulb and internal carotid artery (ICA) is a more accurate predictor for coronary artery disease than mean and maximum IMT [5]. But, for patients with cerebrovascular disease and ICA stenosis it is still unknown.

CCA-IMT progression is commonly used as surrogate endpoint for cardiovascular risk for evaluating drug therapy in interventional studies [6–9]. However, CCA-IMT is affected by the dynamic range and frequency bandwidth of the ultrasound system employed [10], while for an elderly subject population image quality is generally poorer than for young healthy subjects. As a consequence the observed IMT distribution is subject to large relative errors. Moreover, CCA-IMT measures vary across studies [11], e.g., mean or maximal CCA-IMT with or without CCA plaque. According to the Mannheim consensus [12], plaques are defined as having a wall thickness 1) extending more than 500 μm into the lumen, 2) and higher than 50% of surrounding IMT and/or 3) higher than 1500 μm. Therefore, the Mannheim criteria use absolute maximal wall thickness (criterion 3) or wall thickness normalized to surrounding IMT (criterion 2) or a combination of both (criterion 1). Because IMT values are slightly higher than the ultrasound resolution (about 0.3 mm for commonly used ultrasound systems), normalization of maximum wall thickness with respect to the surrounding IMT (criterion 2) will introduce wide variations. Considering the interrelationship between wall thickness and artery diameter according to the Lamé’s equation [13, 14] and the wider range in CCA diameter (6–9 mm) in a healthy population [15], it seems physiologically more reasonable to normalize absolute maximal wall thickness by diameter. Using local wall thickness normalized to either IMT (i.e., thickness-to-IMT ratio) or diameter (i.e., thickness-to-diameter ratio) instead of the CCA-IMT as surrogate endpoint may affect the interpretation of drug therapy results. In addition, normalized wall thickness may lead to reclassification of CCAs towards another risk category, e.g. risk of having more than 50% degree of ICA stenosis.

This study analyses the baseline results of a 2-year follow-up PARISK study in which the association between CCA wall parameters and risk of plaque rupture will be investigated [16]. As a first step, we will investigate the interrelationship of CCA-IMT parameters and their association with the degree of ipsilateral ICA stenosis. More specifically, we will investigate in a large group of symptomatic subjects 1) the relevance of absolute and normalized maximal wall thickness with or without CCA plaques, 2) their relation with CCA-IMT inhomogeneity and 3) the association between absolute wall thickness, thickness-to-diameter ratio, thickness-to-IMT ratio, CCA plaques and CCA-IMT inhomogeneity with the degree of ipsilateral ICA stenosis.

## Methods

### Study subjects

240 patients with mild-to-moderate ICA stenosis (<70% according to the NASCET criteria) and recent ischemic stroke, transient ischemic attack or amaurosis fugax, were included in the Plaque At RISK (PARISK) study (clinical trials.gov NCT01208025), an ongoing multicenter cohort study with 2-year follow-up. Details of the study were previously described [16]. The study was approved by the Medical Ethics Committees of the participating centers and all patients gave written informed consent. Currently, only baseline observations are available.

### Data acquisition

Longitudinal ultrasound B-mode recordings (40 mm width, 5 s, 37 fps) of both CCAs were acquired in duplicate of 233 patients at anterolateral and posterolateral angles with a Philips iU22 scanner (Philips Medical Systems, Bothell, USA) using different probes (17–5, 12–5 or 9–3 MHz) depending on the CCA depth. The distal end of the recorded CCA segment was located 1–2 cm proximal to the flow divider. During ultrasound recordings, patients lay in supine position with their head slightly tilted to the opposite side. Due to contra-indications (low renal clearance (<60 ml/min) or allergy to CT contrast media), only 201 patients were subjected to multidetector computed tomography angiography (MDCTA).

### Echo edge detection

Wall thickness was extracted at end-diastole by edge detection of B-mode images with dedicated software developed by Maastricht University Medical Center (MUMC, Maastricht, The Netherlands) [17] by a trained observer blinded to the MDCTA results. The intra-subject precision of the adopted software for absolute IMT of an artery segment, i.e. the standard deviation of differences between duplicate recordings and their average, is on average 99 μm [1]. The maximum variation of IMT expected due to the ultrasound depth resolution is 150 μm [1]. For each B-mode frame, automatic wall detection of the media-adventitia transition at the anterior and posterior wall was performed for half-overlapping segments (width 3.7 mm) using a threshold of 65% (or half of the difference between this threshold and the maximum, i.e., 83%, in case of an echogenic lumen-intima boundary) of the maximal grey value of the adventitia segment [1]. The local diameter was defined as the local difference along the ultrasound beam between anterior and posterior media-adventitia transitions.

The diameter waveforms, as extracted by edge detection, were smoothed over time (0.2 s filter span) with a 2nd order zero-phase Savitsky-Golay filter. After discarding the end-segments, the mean diameter waveform was calculated and the end-diastolic frames identified. At those frames, the lumen-intima transition along the posterior wall was identified, based on the maximum of the first derivative of the echo amplitude, and corrected manually when necessary [1]. The spatial IMT distribution was obtained as the differences along the ultrasound beam between the posterior lumen-intima and media-adventitia transitions over the artery segment.

### Absolute and normalized maximal wall thickness

For each end-diastolic image, the diameter and IMT were obtained as the spatial median while the maximal wall thickness as spatial maximum, and averaged (median) over all available heart beats (on average 5). Absolute maximal wall thickness was normalized to the median end-diastolic diameter, defined as thickness-to-diameter ratio, and to the median IMT, defined as thickness-to-IMT ratio. All parameters were averaged (median) over all ipsilateral recordings.

### IMT inhomogeneity

Absolute IMT inhomogeneity was defined as the standard deviation of the IMT over the artery segment and averaged (median) over available heart beats and all ipsilateral recordings (anterolateral, posterolateral, duplicate). IMT inhomogeneity was also normalized to the local end-diastolic diameter, i.e., relative IMT inhomogeneity.

### Degree of ICA stenosis

MDCTA images were analyzed with dedicated 3D analysis software (Leonardo and syngo.via; Siemens, Erlangen, Germany). Degree of stenosis in both carotid arteries (bifurcation or ICA), based on the European Carotid Surgery Trial criteria [18], was manually assessed perpendicularly to the central lumen line by a trained observer.

### Statistical analysis

To compare the maximal wall thickness parameters, the parameters were transformed to a normal z-score distribution using the expression (value-mean)/SD. The mean and standard deviation (SD), used as reference in this equation, were derived for CCA arteries without plaques according to the Mannheim criteria. To compare maximal wall thickness parameters with IMT inhomogeneity, correlation coefficients were calculated.

Optimal cut-offs for absolute maximal wall thickness, thickness-to-diameter ratio and thickness-to-IMT ratio for the presence of a >50% ipsilateral stenosis were derived from ROC curves. The optimal cut-off follows from the shortest distance towards the upper left corner of the ROC curve. In addition, a Student t-test was used to assess the difference in degree of ICA stenosis for ipsilateral CCA arteries with low and high wall thickness parameters.

To establish the risk for having more than 50% degree of ipsilateral ICA stenosis, the CCAs were divided into low and high absolute maximal wall thickness, thickness-to-diameter ratio and thickness-to-IMT ratio according to the ROC defined cut-offs. Since the variation in IMT due to the ultrasound depth resolution (conservatively estimated at 300 μm) is about 150 μm, i.e. 2% for an end-diastolic diameter of 7.5 mm, the cut-off level for relative IMT inhomogeneity was tentatively set at 2% [1]. To investigate the effect of wall thickness parameters as risk markers on the defined risk categories, reclassification of arteries was defined as the number of CCAs that switched to another risk category, using either the maximal wall thickness parameters instead of the Mannheim criteria or thickness-to-diameter ratio instead of absolute maximal wall thickness. Values are quantified as mean ± SD. Significance level was set at *p* < 0.05.

## Results

In total, 197 patients received an MDCTA as well as an ultrasound examination. Five patients were excluded due to insufficient quality of MDCTA (*N* = 2) or due to failure to have an ultrasound registration of both CCAs (*N* = 3). In addition, patients with an ICA occlusion or stent (*N* = 3) were excluded, leading to 189 included patients (371 CCAs; mean age 68 ± 9 years). Patient characteristics are shown in Table 1. Prior to the study we estimated the B-mode depth resolution both from the spatial speckle frequency (ensemble average power spectral density across image) and from the width at half-maximum of distinct lumen-intima echoes (average of 10 independent observations) at 264 and 267 um, respectively.

**Table 1:** Patient characteristics. Data are presented as mean ± SD (range or number of patients)

Number	189	-
Age	68 ± 9 (39–88)	years
Male	73 (*N* = 138)	%
BMI	27 ± 4 (17–43)	kg/m^2^
Systolic blood pressure	140 ± 19 (97–210)	mmHg
Diastolic blood pressure	79 ± 9 (54–105)	mmHg
Pulse pressure	61 ± 16 (27–117)	mmHg
Stroke / TIA/ amaurosis fugax	46/42/12 (*N* = 87/80/22)	%
Current smoking	23 (*N* = 43)	%
Diabetes Mellitus	21 (*N* = 41)	%
Hypercholesterolemia	57 (*N* = 107)	%
Hypertension	59 (*N* = 111)	%

### Absolute and normalized maximal wall thickness

**Fig. 1:**
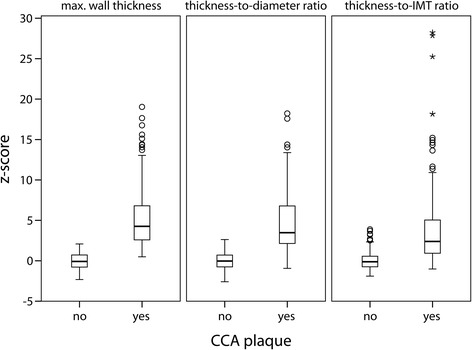
Absolute maximal wall thickness, thickness-to-diameter ratio and thickness-to-IMT ratio of the CCA as function of the presence of CCA plaque. Values are presented as normal z-scores, based on the mean and SD of the thickness parameters for arteries without CCA plaques. Arteries with CCA plaques clearly have a significantly larger wall thickness. Normalized thickness-to-IMT has a wider distribution than maximal wall thickness and thickness-to-diameter ratio

Figure 1 contains a boxplot of absolute and normalized maximal wall thickness, expressed as normal z-scores. CCA arteries with plaques (*N* = 140) according to the Mannheim criteria clearly have a higher absolute maximal wall thickness, thickness-to-diameter ratio and thickness-to-IMT ratio than CCAs without plaques (*N* = 231; mean difference 5, 5 and 4, respectively, Student t-test *p*-value <0.001). The values of the thickness-to-IMT ratio of CCAs with plaques are spread over a wider range than the other wall thickness parameters (Fig. 1) due to resolution related variations. Therefore, thickness-to-IMT ratio is not considered for correlation with IMT inhomogeneity.

### Maximal wall thickness and IMT inhomogeneity

**Fig. 2:**
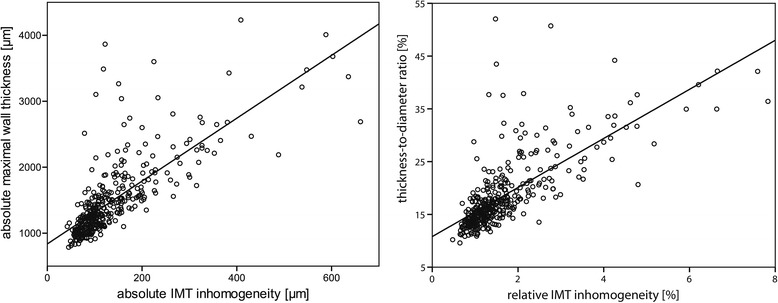
Absolute maximal wall thickness as function of absolute IMT inhomogeneity (*left*) and thickness-to-diameter ratio as function of relative IMT inhomogeneity (*right*). A strong correlation exists between absolute maximal wall thickness and absolute IMT inhomogeneity (*R* = 0.76) and between thickness-to-diameter ratio and relative IMT inhomogeneity (*R* = 0.73)

Absolute maximal wall thickness is strongly correlated with absolute IMT inhomogeneity (*R* = 0.76, Fig. 2). In addition, maximal thickness-to-diameter ratio is also strongly associated with relative IMT inhomogeneity (*R* = 0.73, Fig. 2).

### Maximal wall thickness and degree of ipsilateral ICA stenosis

**Fig. 3:**
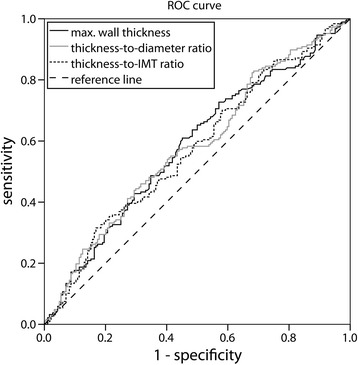
ROC curve for absolute maximal wall thickness (*black line*), thickness-to-diameter ratio (*grey line*) and thickness-to-IMT ratio (*dotted line*) for determination of a >50% ipsilateral ICA stenosis. Optimal cut-off values with the shortest distance (0.60, 0.61 and 0.64, respectively) towards the left upper corner are 1277 μm for absolute maximal wall thickness, 17% for thickness-to-diameter ratio and 129% for thickness-to-IMT ratio

ROC curves of absolute and normalized maximal wall thickness for detecting an ipsilateral ICA stenosis greater than 50% are shown in Fig. 3. Optimal cut-offs for absolute maximal wall thickness, thickness-to-diameter ratio and thickness-to-IMT ratio are 1277 μm, 17% and 129%, respectively. When only the side with the highest ICA plaque is considered, optimal cut-offs are 1191 μm, 16 and 124%, respectively.

### Risk stratification

Arteries were divided into two risk categories according to the ROC defined cut-offs for an ICA stenosis (see above). When thickness-to-IMT ratio instead of Mannheim criteria is used as risk marker, a large number of arteries (Table 2, *N* = 95, 26%) are reclassified to another risk category. Moreover, thickness-to-diameter ratio reclassifies 53 arteries (14%), whereas absolute maximal wall thickness reclassifies 56 arteries (15%). Since thickness-to-IMT ratio is more prone to resolution related variations (Fig. 1), only absolute maximal wall thickness and thickness-to-diameter ratio are considered for further analyses.

**Table 2:** Number of arteries with low or high maximal wall thickness parameters, stratified according to CCA plaque presence (Mannheim criteria). Using maximal wall thickness parameters as risk markers instead of Mannheim criteria results in reclassification of subjects towards another risk category. For example, the thickness-to-IMT ratio (right columns) reclassifies 70 and 25 subjects towards a higher and lower risk category, respectively, in total 26%

CCA plaque	Absolute maximal wall thickness		Thickness-to-diameter ratio		Thickness-to-IMT ratio	
	<1277 μm	>1277 μm	<17%	>17%	<129%	>129%
No	176	55	185	46	161	70
Yes	1	139	7	133	25	115
Total	177	194	192	179	186	185

**Fig. 4:**
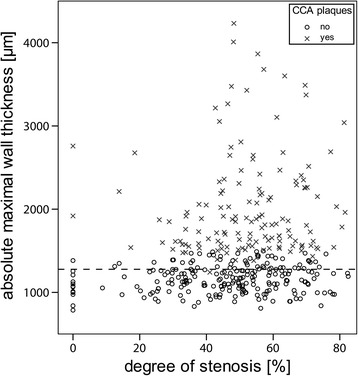
Absolute maximal wall thickness of the CCA as function of degree of ipsilateral ICA stenosis. Patients with absolute maximal wall thickness below the ROC defined cut-off (*dashed line*) have a wide range of plaque sizes whereas patients with absolute maximal wall thickness above the ROC defined cut-off have larger degree of ICA stenosis

Patients with absolute maximal wall thickness below 1277 μm have a wide range of degree of ipsilateral ICA stenosis (*N* = 174), whereas patients with absolute wall thickness above 1277 μm (*N* = 197) exhibit a higher degree of ICA stenosis (Fig. 4). Both absolute maximal wall thickness and thickness-to-diameter ratio above cut-off values are associated with a higher ipsilateral stenosis degree (Tables 3 and 4, 52 ± 15% and 52 ± 16%, respectively) than below the cut-off values (mean difference 8% and 7%, Student t-test *p*-value <0.001). This association remains borderline significant after excluding patients with CCA plaques (Tables 3 and 4, mean difference 7% and 5%, Student t-test *p*-value 0.02 and 0.15 respectively). Moreover, similar trends are seen when only the side with the highest ICA plaque is considered (Tables 3 and 4). In addition, CCAs with a plaque (*N* = 140) exhibit a stronger association with a higher distal stenosis degree than arteries without a plaque (*N* = 231; 52 ± 15% and 46 ± 19%, respectively, mean difference 7%, Student t-test *p*-value < 0.001). When thickness-to-diameter ratio instead of absolute maximal wall thickness is used as risk marker for having more than ipsilateral 50% degree of ICA stenosis, 55 CCAs (15%) are reclassified towards the other risk category (of which *N* = 20 towards a higher category).

**Table 3:** ICA stenosis degree according to the ipsilateral absolute maximal wall thickness cut-off for ICA stenosis at either side or for the largest ICA stenosis. Data are presented as mean ± SD. A high absolute maximal wall thickness is indicative for a higher degree of ipsilateral ICA stenosis

ICA Plaque	N ICAs	CCA plaque	Cut-off	ICA stenosis	*p*-value
Either side	174	yes/no	<1277 μm	44 ± 20%	<0.001
	197		>1277 μm	52 ± 15%	
	173	no	<1277 μm	44 ± 20%	0.02
	58		>1277 μm	51 ± 16%	
Largest	64	yes/no	<1191 μm	55 ± 14%	0.006
	121		>1191 μm	60 ± 11%	
	64	no	<1191 μm	55 ± 14%	0.04
	54		>1191 μm	60 ± 12%

**Table 4:** ICA stenosis degree according to the ipsilateral thickness-to-diameter ratio cut-off for ICA stenosis at either side or for the largest ICA stenosis. Data are presented as mean ± SD. A high thickness-to-diameter ratio is indicative for a higher degree of ipsilateral ICA stenosis

ICA plaque	N ICAs	CCA plaque	Cut-off	ICA stenosis	*p*-value
Either side	192	yes/no	<17%	45 ± 19%	<0.001
	179		>17%	52 ± 16%	
	185	no	<17%	45 ± 19%	0.15
	46		>17%	50 ± 18%	
Largest	91	yes/no	<16%	56 ± 13%	0.03
	94		>16%	61 ± 12%	
	90	no	<16%	56 ± 13%	0.3
	23		>16%	58 ± 12%	

### IMT inhomogeneity and degree of ipsilateral ICA stenosis

Arteries with a relative IMT inhomogeneity above 2% (*N* = 81) are associated with a higher degree of ipsilateral ICA stenosis (53 ± 11%) than arteries (*N* = 290) with a relative IMT inhomogeneity below 2% (mean difference 6%, Student t-test *p*-value <0.001).

## Discussion

We evaluated the absolute and normalized maximal wall thickness and CCA-IMT inhomogeneity in patients with a recent cerebrovascular accident and mild-to-moderate ICA stenosis. Normalization by median IMT leads to large variations. Absolute maximal wall thickness and thickness-to-diameter ratio are strongly correlated with absolute and relative IMT inhomogeneity, respectively. IMT inhomogeneity does not provide extra information on top of absolute maximal wall thickness or thickness-to-diameter ratio in relation to the degree of ipsilateral ICA stenosis. Mainly CCA plaques are strongly associated with a higher degree of ipsilateral ICA stenosis. Although a similar trend is seen for both absolute maximal wall thickness and thickness-to-diameter ratio, 15% of CCAs are reclassified when thickness-to-diameter ratio instead of absolute maximal wall thickness is used as risk marker for a >50% ipsilateral ICA stenosis.

Maximal wall thickness is normalized by the median end-diastolic diameter as well as the median IMT. As expected, patients with CCA plaques have a significantly higher absolute and normalized maximal wall thickness (p < 0.001). Normalization by the median IMT leads to similar values for arteries without CCA plaques, whereas a large variation is found for CCA arteries with large plaques (Fig. 1).

### Risk stratification

Optimal cut-offs for absolute and normalized maximal wall thickness are derived from ROC curves for a >50% ipsilateral ICA stenosis (Fig. 3). We have chosen those plaques since the smaller plaques in the curved carotid bulb hardly induce hemodynamic changes [19, 20]. 96 CCAs (Table 2; 26%) are reclassified towards another risk category when thickness-to-IMT ratio rather than the Mannheim criteria are used as a risk marker for a >50% ipsilateral ICA stenosis. Since observed IMT values are generally noisy because they are slightly higher than the ultrasound depth resolution, normalization of maximum wall thickness with respect to median IMT will introduce wide variations. Therefore, it is questionable whether the Mannheim criterion [12], defining a plaque if the maximum thickness is 50% greater than the surrounding IMT, is consistent. Moreover, “surrounding IMT” is quite arbitrary (where does a plaque begin or end); that is why we decided to normalize the maximum thickness by the median IMT.

For the risk stratification of individual patients an optimal cut-off is needed. Although maximal wall thickness and thickness-to-diameter ratio show similar associations with the degree of ipsilateral ICA stenosis (Table 3 and 4), 55 arteries (15%) are reclassified when thickness-to-diameter ratio instead of absolute maximal wall thickness is used as risk marker for a >50% ipsilateral ICA stenosis. Alternately, an age dependent cut-off for absolute maximal wall thickness may be considered to correct for differences in CCA diameter [15, 21]. However, our population has a wide diameter distribution (8083 ± 1048 μm; range 5769–11702 μm), which cannot be explained by age differences only (68 ± 9 years; range 39–88 years). Since the vessel diameter depends on subject size, and diameter and wall thickness are interrelated via the Lamé’s equation [13, 14], it seems more reasonable to use normalized rather than absolute maximal wall thickness values.

### Wall thickness parameters and ipsilateral stenosis degree

High relative IMT inhomogeneity and high thickness-to-diameter ratio are both associated with a higher degree of ICA stenosis (mean difference 6% and 7%, respectively, Student t-test *p*-value < 0.001). Since we look at local CCA features, the current *p*-value is lower than observed when the average relative IMT inhomogeneity of both CCAs is considered [1]. Because maximal wall thickness and thickness-to-diameter ratio are highly correlated with absolute and relative IMT inhomogeneity (Fig. 2) and similar trends are observed in relation to degree of stenosis, absolute or relative IMT inhomogeneity does not provide extra information on top of maximal wall thickness or thickness-to-diameter ratio. Mainly the presence of a CCA plaque dominates the association with a higher degree of ICA stenosis (mean difference 7%, Student t-test *p*-value < 0.001).

### Plaques and wall thickness

Almost all CCAs with plaques according to the Mannheim consensus have a high maximal wall thickness and thickness-to-diameter ratio according to the cut-off derived with the ROC. Our population has a high incidence of CCA plaques (140 of 371 arteries; 38%). The presence of CCA plaques is rare in a healthy population, male 6% and female 3% [22], and is more prevalent (22%) in older subjects (>65 years) [23]. The relatively high incidence of CCA plaques in our population is attributable to the fact that subjects exhibited cerebrovascular symptoms and, therefore, belong to a diseased population.

It is questionable whether IMT and plaque formation are driven by the same process. IMT is strongly associated with hypertension and age [24] and is inheritable [25–27]. However, the heritability of plaque is less strong [27] and attributed to various genes [27–29]. Furthermore, since the intima thickness is approximately only 0.02 mm [30], IMT is mainly affected by hypertensive medial hypertrophy [31] whereas atherosclerosis is an inflammatory process where plaque formation starts with pathological intimal thickening and lesions containing lipid pools [32]. Therefore, IMT and plaque formation are likely different phenotypes [33–35]. Our study shows that the association between ICA stenosis and ipsilateral CCA plaques is highly significant (Table 3), which is in line with the concept of atherosclerosis as a more widespread instead of a focal disease, prompting a global rather than a focused vascular examination. Whether elevated CCA wall parameters are present before development of an ICA stenosis cannot be established in our study.

## Conclusion

In conclusion, to evaluate wall thickness it is more reasonable to normalize maximal wall thickness by end-diastolic diameter rather than by IMT, suggesting a modification of the Mannheim criteria. Absolute or relative IMT inhomogeneity does not provide extra information on top of maximal wall thickness or thickness-to-diameter ratio. Mainly CCA plaques are strongly associated with a higher degree of ipsilateral ICA stenosis. Although a similar trend is seen for both absolute maximal wall thickness and thickness-to-diameter ratio, 55 arteries (15%) are reclassified when thickness-to-diameter ratio instead of absolute maximal wall thickness is used as risk marker for a >50% ICA stenosis. Whether this reclassification is clinically important and relative IMT inhomogeneity and thickness-to-diameter ratio have predictive value for plaque progression and cerebrovascular events will be evaluated in the follow-up phase of the PARISK study.

## Abbreviations

CCA: Common carotid artery
CT: Computed tomography
ICA: Internal carotid artery
IMT: Intima-media thickness
MDCTA: Multidetector computed tomography angiography
MUMC: Maastricht University Medical Center
PARISK: Plaque At RISK
SD: Standard deviation
